# MRI Tracking of SPIO- and *Fth1*-Labeled Bone Marrow Mesenchymal Stromal Cell Transplantation for Treatment of Stroke

**DOI:** 10.1155/2019/5184105

**Published:** 2019-08-29

**Authors:** Xiaolei Huang, Yang Xue, Jinliang Wu, Qing Zhan, Jiangmin Zhao

**Affiliations:** ^1^Shanghai Baoshan Hospital of Integrated Traditional Chinese and Western Medicine, Shanghai 201900, China; ^2^Shanghai 6th People's Hospital, Shanghai Jiao Tong University, Shanghai 200233, China; ^3^Tianjin Medical University Hospital for Metabolic Diseases, Tianjin 300070, China; ^4^Department of Neurology, Seventh People's Hospital of Shanghai University of TCM, Shanghai 200137, China; ^5^Department of Medical Imaging, Shanghai 9th People's Hospital, Shanghai Jiao Tong University School of Medicine, Shanghai 200010, China

## Abstract

We aimed to identify a suitable method for long-term monitoring of the migration and proliferation of mesenchymal stromal cells in stroke models of rats using ferritin transgene expression by magnetic resonance imaging (MRI). Bone marrow mesenchymal stromal cells (BMSCs) were transduced with a lentivirus containing a shuttle plasmid (pCDH-CMV-MCS-EF1-copGFP) carrying the ferritin heavy chain 1 (*Fth1*) gene. Ferritin expression in stromal cells was evaluated with western blotting and immunofluorescent staining. The iron uptake of *Fth1*-BMSCs was measured with Prussian blue staining. Following surgical introduction of middle cerebral artery occlusion, *Fth1*-BMSCs and superparamagnetic iron oxide- (SPIO-) labeled BMSCs were injected through the internal jugular vein. The imaging and signal intensities were monitored by diffusion-weighted imaging (DWI), T2-weighted imaging (T2WI), and susceptibility-weighted imaging (SWI) *in vitro* and *in vivo*. Pathology was performed for comparison. We observed that the MRI signal intensity of SPIO-BMSCs gradually reduced over time. *Fth1*-BMSCs showed the same signal intensity between 10 and 60 days. SWI showed hypointense lesions in the SPIO-BMSC (traceable for 30 d) and *Fth1*-BMSC groups. T2WI was not sensitive enough to trace *Fth1*-BMSCs. After transplantation, Prussian blue-stained cells were observed around the infarction area and in the infarction center in both transplantation models. *Fth1*-BMSCs transplanted for treating focal cerebral infarction were safe, reliable, and traceable by MRI. *Fth1* labeling was more stable and suitable than SPIO labeling for long-term tracking. SWI was more sensitive than T2W1 and suitable as the optimal MRI-tracking sequence.

## 1. Introduction

Magnetic resonance imaging (MRI) is an important tool for cellular imaging as it can be used for clinical diagnostics. Current therapies for disorders due to ischemia reperfusion are unable to repair damaged or lost neural cells because neural cells are nonproliferative cells and limited in number. Bone marrow mesenchymal stromal cells (BMSCs) have been used for several regenerative methods in animal models or patients [1]. Nevertheless, the fate of transplanted BMSCs in live animals is still poorly understood [2–4]. Thus, a noninvasive, real-time, sensitive, and clinically applicable method for tracking transplanted BMSCs and monitoring their behavior in live animals would be useful. Superparamagnetic iron oxide (SPIO) has been used in several studies for tracking BMSCs *in vitro* and in *vivo* [1, 5, 6]. However, the hypointense MRI signal generated by these particles is not sustainable over a long time because the iron oxide nanoparticles are diluted with each cell division. Moreover, SPIO nanoparticles can be targeted and cleared by macrophages [7, 8]. Consequently, BMSCs labeled with SPIO may not be a suitable method for long-term tracking of BMSC engraftment. Tracking reporter genes which express MRI-detectable proteins are a newly developing approach to monitor transplanted cells [9]. Ferritin, a ubiquitously expressed protein *in vivo*, can be visualized by MRI when overexpressed and can store iron in a nontoxic manner [7, 10–12]. In our study, we utilized a lentivirus carrying the ferritin heavy chain 1 (*Fth1*) gene [13], which encodes the ferritin protein to tag BMSCs, and then tracked the survival, migration, and proliferation of transplanted cells over a period of time.

## 2. Materials and Methods

### 2.1. Culture and Identification of BMSCs

All interventions and animal care procedures were performed in accordance with the Laboratory Animal Welfare Act, Guide for the Care and Use of Laboratory Animals (National Institutes of Health, Bethesda, MD), and Guidelines and Policies for Animal Surgery provided by our hospital (Shanghai Jiaotong University, Shanghai, China) and were approved by the Institutional Animal Use and Care Committee.

BMSCs were isolated and cultured from the whole bone marrow of Sprague Dawley (SD) rats using the direct adherence method. The culture medium was replaced after the first 8 h and then changed every other day. We chose cells passaged for 3–5 generations for our experiments. To determine whether BMSCs expressed the surface marker, flow cytometric analysis of BMSCs was performed five independent times. In brief, the cells were incubated with PE-CD34, PE-CD44, and FITC-CD90 (AbD Serotec, Germany) antibodies for 30 min. Fluorescence-associated cell sorting was performed using a BD LSR II benchtop analyzer (BD Biosciences, America) for flow cytometry. To evaluate whether the BMSCs possessed differentiation capability, BMSCs were treated with [1] induction media (Cyagen, Guangzhou, China) to test the differentiation capability. The morphology change in BMSCs was analyzed.

### 2.2. Vector Design and Viral Production

*Fth1* was amplified by PCR from the genomic DNA of rats to construct the overexpression lentiviral vector pCDH-CMV-MCS-EF1-copGFP-*Fth1*. The open reading frame (ORF) of *Fth1* was designed to contain the restriction enzyme sites needed for cloning. For viral production, the transfer plasmid pCDH-CMV-MCS-EF1-copGFP-*Fth1* (expressing the proteins gag, env, and FTH1) was transiently cotransfected with the packaging plasmid psPAX2 (Addgene 1226, expressing the proteins gag and pol) and the envelope plasmid pMD2.G (Addgene 12259, expressing the protein VSV-G). The primer sequences used are as follows:

1. pCDH-*Fth1*-F: 5′-AATGAATTCGCCACCATGACCACCGCGTCTCCCTCGC-3′
2. pCDH-*Fth1*-R: 5′-AACGGATCCTTAGCTCTCATCACCGTGTCCC-3′

The red region shows the restriction enzyme cutting site. Supplemental [Supplementary-material supplementary-material-1] shows the features of the three vectors.

*Fth1* was introduced into the lentiviral vector pCDH-CMV-MCS-EF1-copGFP and then transduced to establish a clonal transgenic line of BMSCs. The following day, Dulbecco's modified Eagle's medium (DMEM)/F-12 medium was replaced with the viral medium. After 48 d, positive colonies were assessed mainly by GFP expression. Lentiviral vectors are supposed to be one of the most effective and stable methods to introduce a transgene, in both primate and murine cells [14]. Total RNA was extracted from HEK-293T cells and utilized for producing a cDNA pool.

### 2.3. BMSC Culture and Lentiviral Transduction

Three milliliters of the supernatant of *Fth1* lentivirus were mixed with an equal volume of the complete medium (DMEM/F-12 supplemented with 15% fetal bovine serum), which served as a lentiviral transduction compound medium. Before lentiviral transduction, 3^rd^ or 4^th^ passage BMSCs were grown to 80%∼90% confluence, and then 3 × 10^5^ cells were cultured for 24 h with the compound medium. After that, the compound medium was replaced with the DMEM/F-12 medium. The transduction efficiency was assessed by the expression of EGFP after 3-4 d. The *Fth1*-BMSCs with EGFP expression efficiency over 70% were maintained and propagated for subsequent experiments.

### 2.4. Fluorescence Quantitative Real-Time PCR

Fluorescence quantitative real-time PCR (SYBR Premix Ex Taq, TaKaRa, JP) was used to assess the expression of the reporter gene *Fth1*. The reaction program was set as 95°C/30 s, 40 cycles of 95°C/5 s, 95°C/15 s, 60°C/15 s, 70°C/10 s, and then cooling at 4°C. We analyzed the relative gene expression data using the 2^−ΔΔCT^ method.

### 2.5. Western Blot

In order to assess the protein levels produced by translation of the *Fth1* mRNA, BMSCs were transfected with the *Fth1* lentivirus, and after 7 d, they were lysed in the prechilled lysis buffer (without bromophenol blue) with 50 *μ*l PMSF (10 mg/ml) and homogenized with a sonicator. Proteins were extracted, quantified, and run on a gel using an SDS-PAGE system (Tianneng Company, Shanghai, China). Anti-ferritin heavy chain 1 (Santa Cruz, USA) was used as the primary antibody. Goat Anti-Rabbit IgG/AP (Santa Cruz, USA) was used as the secondary antibody. The relative expression of ferritin in BMSCs was analyzed from the band intensity using the Quantity One software (Bio-Rad), with normalization to GAPDH (Cell Signaling Technology, USA) (dilution 1 : 1000).

### 2.6. Evaluation of the Intracellular Iron Concentration and Cytotoxicity of *Fth1* Expression by CCK8 and Prussian Blue Iron Staining

In order to evaluate the concentration of supplemented iron that the BMSCs can tolerate, the viability and intracellular iron accumulation of the cells were qualitatively assessed with Cell Counting Kit-8 (CCK8) and Prussian blue iron staining after 48 h of exposure to a range of iron concentrations. To find out the optimum ferric ammonium citrate concentration cultured with *Fth1*-BMSCs while preparing for in vitro MRI examination, we cultured 3, 4, 5, and 7 mmol/ml ferric ammonium citrate with *Fth1*-BMSCs in the preliminary experiment, and considering cytotoxic effects and iron concentrations, we chose 5 mmol/ml ferric ammonium citrate for the next step. Fifth- or sixth-passage *Fth1-*BMSCs were plated at a density of 10000 cells/well, cultured in 96-well plates with 5 mmol/ml ferric ammonium citrate for 24 h, and then cultured for further 48 h. The absorbance of *Fth1-*BMSCs at 450 nm was measured using the Epoch Multi-Volume spectrometer system (BioTek Instruments, Winooski, VT, USA) after incubation for 1 h/2 h. All experiments were independently performed 5 times.

The average intracellular iron accumulation and distribution in the BMSCs were qualitatively assessed with Prussian blue iron staining. Fifth- or sixth-passage *Fth1-*BMSCs grown on coverslips were washed with PBS, fixed with 4% paraformaldehyde for 20 min, and stained with Prussian blue for 20 min; the cell nuclei were counterstained with nuclear fast red solution for 10 min. The positive and blank controls contained 50 *μ*g/ml SPIO-BMSCs and BMSCs, respectively. The coverslips were mounted and examined under a microscope (Olympus BX51).

### 2.7. *In Vitro* MRI of *Fth1*-BMSCs

Fifth- to sixth-passage *Fth1-*BMSCs cultured in plates with an optimum concentration of ferric ammonium citrate for 24 h were washed with PBS to remove excess ferric ammonium citrate. *Fth1-*BMSCs (1 × 10^6^ cells) were embedded in 600 *μ*L PBS and 400 *μ*L agarose (1%) uniformly in 1.5 ml cryotubes and kept for 30 min at room temperature (25°C). The positive and blank controls contained 50 *μ*g/ml SPIO-BMSCs and BMSCs, respectively. We chose a series of survey images as observation indices including T2-weighted imaging (T2WI) and susceptibility-weighted imaging (SWI) sequences using a 1.5 T MRI scanner (General Electric, USA). Scanning parameters were as follows: (1) T2WI a with fast spin echo (FSE) sequence, TR/TE = 2250/102 ms, and depth = 12.5 mm. (2) SWI with a SWAN sequence, TR/TE = 82.8/44.7 ms, flip angle = 15°, and depth = 41.67 mm.

### 2.8. *In Vivo* MRI with *Fth1-*BMSC Transplantation

SD rats (*n* = 16) were chosen to establish focal cerebral infarction models [3] and using a random number table were divided into the control group (*n* = 4), SPIO-BMSC transplantation treatment group (*n* = 6), and *Fth1-*BMSC transplantation treatment group (*n* = 6). *Fth1-*BMSCs (3 × 10^5^ cells) and an equal amount of 50 *μ*g/ml SPIO-BMSCs were mixed with 500 *μ*l PBS separately, and 100 *μ*l was aspirated into an insulin needle. Focal infarction models of middle cerebral artery occlusion (MCAO) were established by inserting a heparinized fish wire. Two of the 14 rats that underwent the operation died, but the deaths were not related to cell infusion. *Fth1-*BMSCs and 50 *μ*g/ml SPIO-BMSCs were injected in the direction of the cranium through the internal jugular vein, and the injection point was clipped with ophthalmic forceps for 5 min to prevent cell leakage. The MRI measurement points were 1 d, 10 d, 20 d, 40 d, and 60 d after transplantation.

For scan imaging of animals, models were scanned using a 1.5 T MRI scanner (General Electric, USA). The animal was placed in a four-channel volume coil (5 cm inner diameter and 8 cm long) (Shanghai Chenguang) and anesthetized using 3% sodium amobarbital via peritoneal injection. We chose a series of survey images as observation indices including T2-weighted imaging (T2WI), diffusion-weighted imaging (DWI), and susceptibility-weighted imaging (SWI) sequences. Scanning parameters were as follows: (1) T2WI with a fast spin echo (FSE) sequence, TR/TE = 2050/80 ms, NEX (number of excitations) = 4, FOV = 10 mm, and depth = 2 mm. (2) SWI with a SWAN sequence, TR/TE = 87.2/44.3 ms, NEX = 1, flip angle = 15°, and depth = 1 mm. (3) DWI with a DW-EPI sequence, TR/TE = 3200/85.9 ms, *b* value = 0 and 800 s/mm^2^, NEX = 4, and depth = 2 mm.

### 2.9. Histology and Immunohistochemistry

To confirm transgene expression after cell transplantation, animals were euthanized upon completion of the MRI scan, and the brains were removed and assessed by Prussian blue staining.

## 3. Results and Discussion

### 3.1. Culture and Identification of BMSCs

Flow cytometry was used to evaluate the purity of BMSCs that were extracted using the adherence method from total bone marrow cells: the percentage of cells that were positive for surface antigens CD44 and CD90 was 99.9% and 99.6%, respectively (*n* ≥ 3). There were only 5.5% cells expressing CD34, which showed that the purity of isolated BMSCs was high (Supplemental [Supplementary-material supplementary-material-1]).

Osteogenic induction culture: around days 12–14, cells exhibited a long spindle-shaped appearance, and scattered nodules could be observed.

Alizarin Red staining was carried out on day 21 of induction, and orange-red-stained nodules could be seen (Supplemental [Supplementary-material supplementary-material-1]).

Adipogenic induction: around day 12, some cells exhibit scattered lipid droplets and rounded cell morphology. After culturing for 21 d, cells were stained with Oil Red O, and many reddish-brown-stained mature adipocytes and immature adipocytes could be seen (Supplemental [Supplementary-material supplementary-material-1]).

### 3.2. Morphological Observation of *Fth1-*BMSCs

Using a bicistronic lentiviral vector containing a fluorescence reporter [9, 15], we were able to confirm the successful integration of the transgenes via sequence analysis. This ensured constitutive expression and translation of the reporter proteins [16, 17]. Because of slow metabolism, the expression of EGFP was slow. The transduction efficiency was assessed after 3-4 days. Hyperintense spots were observed in cells that expressed EGFP using fluorescence microscopy (Supplemental [Supplementary-material supplementary-material-1]). The transduction efficiency was about 80%. The cells grew well and had normal morphology.

### 3.3. Expression of Reporter Gene *Fth1* Assessed by Fluorescence Quantitative Real-Time PCR

After BMSCs were transfected by *Fth1* lentivirus, fluorescence quantitative real-time PCR was used to assess the expression of the reporter gene *Fth1*, by measuring the amount of mRNA transcripts. qRT-PCR data showed that compared to the control group, in the EGFP-*Fth1* group, *Fth1* expression appeared significantly upregulated (Table 1). The FTH1 protein expression in BMSCs 7 d after transfection with the *Fth1* lentivirus and in normal BMSCs (control group) was assessed by western blotting. The results showed that compared to the control group, FTH1 protein expression in the *Fth1-*BMSC group appeared significantly upregulated (Supplemental [Supplementary-material supplementary-material-1]).

### 3.4. Western Blot to Assess the Protein Levels from Translated mRNA

**Figure 1:**
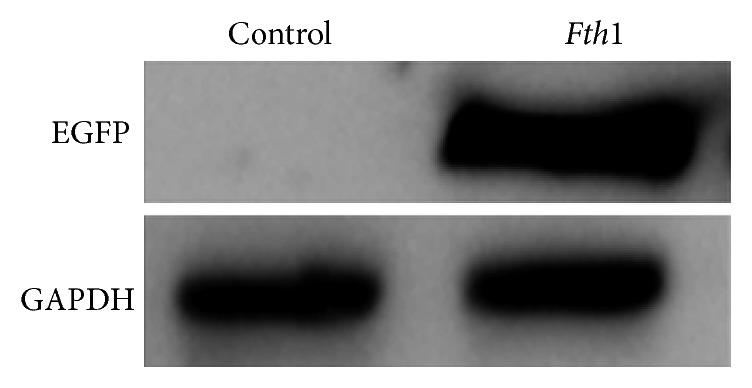
Western blot results of FTH1 expression in the *Fth1*-BMSC group and BMSC group.

The FTH1 protein expression of BMSCs 7 d after transfection with the *Fth1* lentivirus and normal BMSCs (control group) was all assessed by western blotting. The results showed that compared to the control group, FTH1 protein expression in the *Fth1-*BMSC group appeared to be upregulated significantly (Figure 1).

### 3.5. Evaluation of the Cytotoxicity of *Fth1* Expression in BMSCs

**Figure 2:**
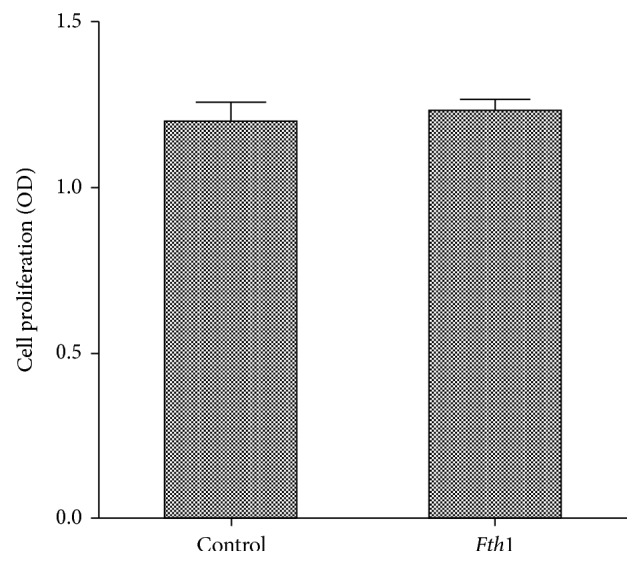
Histogram of the proliferative activity of experimental and control groups detected using the CCK8 method.

The CCK8 method showed that the metabolic activity of the *Fth1-*BMSC group was higher than that of the control group (*P* < 0.05) (Table 2) (Figure 2).

### 3.6. Intracellular Iron Content in *Fth1-*BMSCs

**Figure 3:**
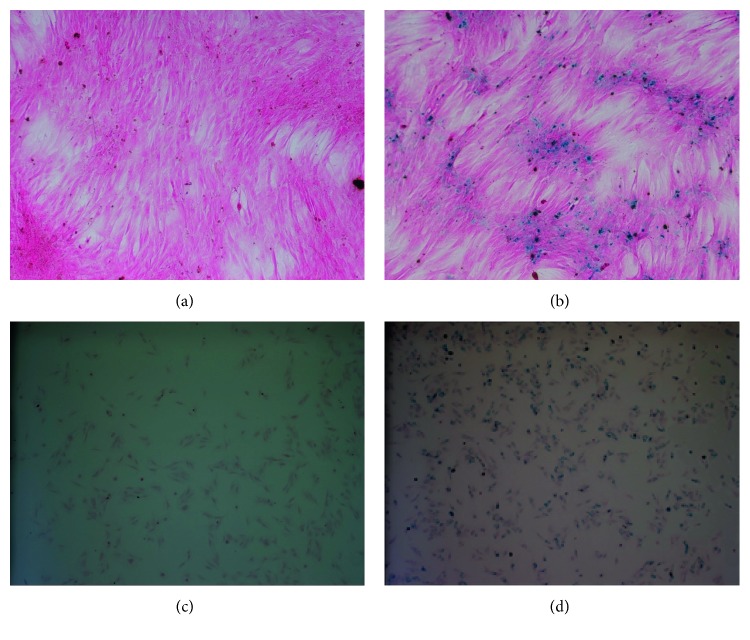
Efficiency of Prussian blue staining in (a, c) BMSCs (×100), where no positively stained cells were seen; (b) *Fth1*-BMSCs (×100), where positively stained cells showed blue-colored masses; (d) SPIO-BMSCs (×100), where positively stained cells showed blue-colored masses.

*Fth1-*BMSCs cultured without ferric ammonium citrate were negative for Prussian blue iron staining. *Fth1-*BMSCs cultured with 5 mmol/ml ferric ammonium citrate showed blue masses, indicating that *Fth1-*BMSCs had a good iron uptake and storage capacity (Figure 3).

### 3.7. *In Vitro* MRI of *Fth1-*BMSCs

**Figure 4:**
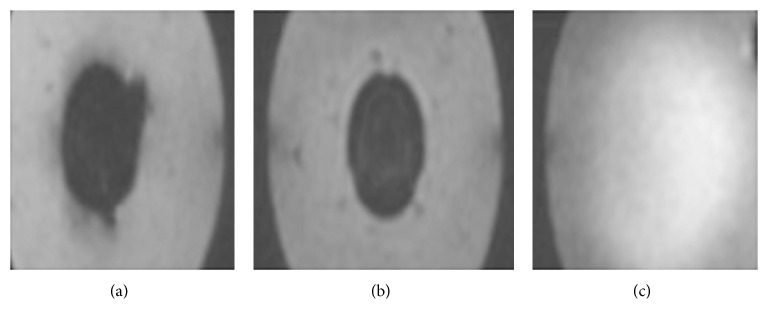
Measurement of cellular iron content and in vitro MRI analysis. MRI images of (a) SPIO-BMSCs showing circular hypointense regions, (b) *Fth1*-BMSCs showing circular hypointense regions, and (c) the control group without obvious hypointense regions.

We observed that the *Fth1-*BMSC suspension showed a good signal contrast in the SWI series but had poor signal contrast in the DWI and T2WI series. Therefore, we chose the SWI sequence to detect the signal changes in the 50 *μ*g/ml SPIO-BMSC and *Fth1-*BMSC groups. The 50 *μ*g/ml SPIO-BMSC and *Fth1-*BMSC groups were significantly hypointense (Figure 4).

### 3.8. *In Vivo* MRI of *Fth1-*BMSCs and SPIO-BMSCs

**Figure 5:**
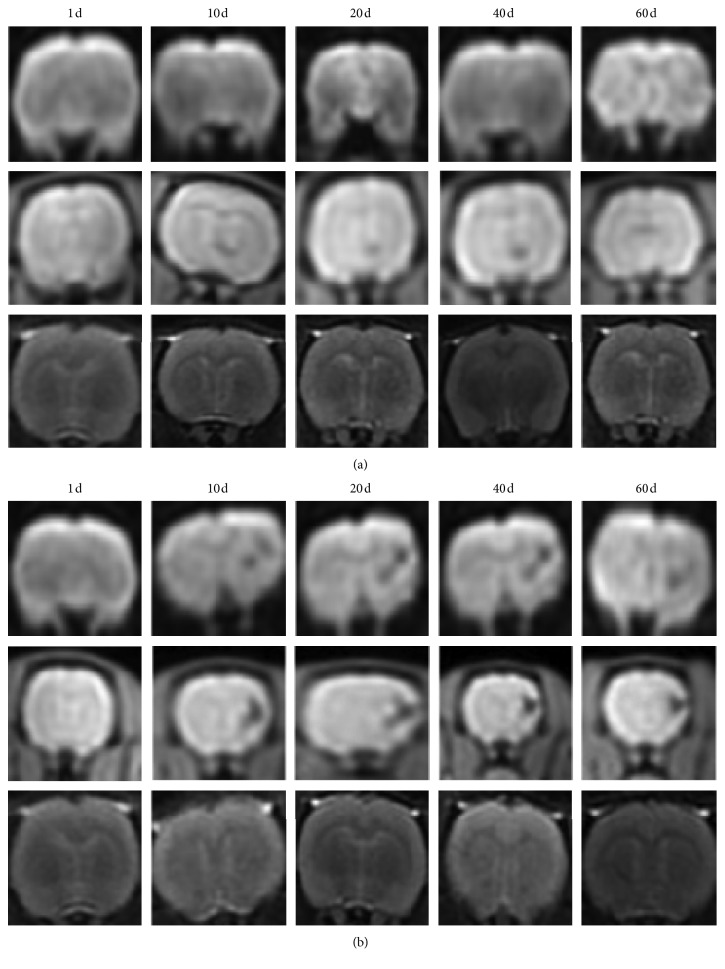
MRI images (DWI, SWI, and T2WI sequences) after transplantation (1 d, 10 d, 20 d, 40 d, and 60 d). (a) SPIO-BMSCs. As time goes on, hypointense signals could still be visible on days 10, 20, and 40 in the SWI sequence. (b) *Fth1*-BMSCs. Hypointense signals could still be visible on days 10, 20, 40, and 60 in the SWI sequence.

After 50 *μ*g/ml SPIO-BMSCs (control group) were injected in the direction of the cranium through the internal jugular vein, we observed well-defined lesions with low signal intensities in the T2WI series around the lateral ventricles of rats after 10 d; the tracing time was about 20 d. *Fth1-*BMSCs were injected in the same way. The MRI measurements were done 1 d, 10 d, 20 d, 40 d, and 60 d after transplantation. No obvious hypointense signal was observed on day 1 but appeared on the right side of the cortex in the DWI and SWI series on day 10. The hypointense signal migrated along nerve fiber tracts to the right side of the infarction lesion in the corpus striatum, representing the chemotaxis and migration of BMSCs. The hypointense signal was steady up to day 60 (Figure 5).

### 3.9. Histology and Immunohistochemistry

**Figure 6:**
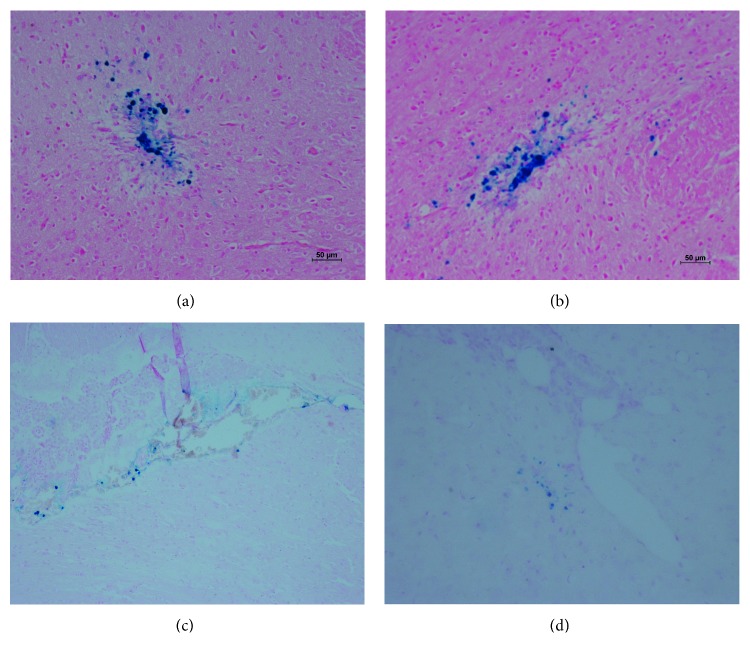
Prussian blue staining of rat brains: positively stained cells showed blue-colored masses. (a, b) *Fth1*-BMSCs (×200). (c, d) SPIO-BMSCs (×200).

The rats were sacrificed after MRI in 60 days using an anesthetic overdose, and the brain was removed; contiguous coronal 12 *μ*m thick sections were prepared for Prussian blue staining. Prussian blue staining was seen on the right side of infarction lesion (Figure 6).

### 3.10. Discussion

We have generated mesenchymal stem cells expressing *Fth1* or labeled with SPIO with the aim of exploring how these iron markers can impact T2WI and SWI contrast using BMSCs. We showed that though we chose a high dose of SPIO (6 × 10^4^/ml) [18], the T2 relaxation time became progressively shorter during *in vivo* MRI tracking of SPIO-BMSCs implanted in the rat cerebral infarcted area probably because of cell mitosis leading to a decrease in the SPIO concentration with each division [8] and because of macrophage phagocytosis, which clears necrotic cells, including SPIO-labeled cells. Since SPIO cells were undetectable in the infarction lesion, we could only perform a pathological analysis. Moreover, the existing technique was too insensitive for tracing low concentrations of iron (SPIO), which affected and limited long-term observation of the biological behavior of BMSCs. Therefore, we used BMSCs transfected with the *Fth1* gene using a lentivirus. The *Fth1*-BMSCs were transplanted to treat focal cerebral infarction induced in rats, and the sensitivity and time duration of signal changes in different MRI sequences were observed, to identify a suitable labeling method for the long-term monitoring of the migration and proliferation of mesenchymal stromal cells.

Ferritin is a ubiquitously expressed intracellular iron-storage protein, keeping iron in a soluble and nontoxic form [19, 20]. The ferritin heavy chain has ferroxidase activity and is responsible for the upregulation of the transferrin receptor, which increases iron uptake. Ferritin heavy chain expression levels showed a linear correlation with the T2 relaxation time [21–23]. *Fth1* can be used as an endogenous reporter gene; when introduced into a lentiviral vector and then used to establish a clonal transgenic BMSC line, the reporter gene can be steadily inherited by the daughter cells during mitosis, indicated by the stable hypointensity observed by MRI [24, 25]. Thus, the *Fth1*-labeling method may be an effective method for long-term monitoring of the biological activity of cells by MRI.

Naumova et al. [16] reported a systematic evaluation of ferritin-labeled stem cells in infarcted mouse hearts *in vivo*, using three cardiac-gated pulse sequences in a 3T scanner. The graft size measurements by T2 iMSDE (improved motion-sensitized driven-equilibrium) and T2 GRE (gradient recalled echo) were highly correlated with histological assessments. These data supported the use of ferritin to track the survival, growth, and migration of stem cells transplanted into the injured heart. Iordanova [17] described an efficient ferritin-based MRI reporter and its use in labeling mouse subventricular-zone progenitors, enabling *in vivo* visualization of endogenous neuroblast migration toward the olfactory bulb. This MRI reporter gene platform can facilitate the noninvasive study of the migration of native or transplanted stem cells and the associated neurogenic or therapeutic molecular events in live animals.

In our *in vitro* experiments, we found that when *Fth1-*BMSCs were cultured in the complete medium, they were well detected by MRI. The ferric ammonium citrate had two main characteristics: first, it assured that *Fth1-*BMSCs could be overexpressed and thus augment iron uptake to reach the detectable threshold of MRI *in vitro*; second, the toxicity of ferric ammonium citrate on cell proliferation could be reduced.

Previous studies have rarely addressed whether FTH1 can be successfully detected by MRI after administration of the *Fth1-*BMSCs and whether *Fth1-*BMSCs can reach the infarction lesions in mouse stroke models when injected through the internal jugular vein [26, 27]. In our *in vivo* experiments, the lacunar stroke model was induced by middle cerebral occlusion. We set up a normal control group, a sham group, and an operation group, and internal jugular vein injection was used to transplant SPIO-labeled BMSCs or *Fth1*-labeled BMSCs. MRI detected BMSCs in the *in vivo* images of different groups. Our present results demonstrate that the SPIO-BMSCs and *Fth1-*BMSCs transplanted by internal jugular vein injection after cerebral ischemia reperfusion injury can be detected *in vivo* and result in significant hypointense signals around the infarction lesion [28]. Statistical analysis showed that SPIO-BMSCs showed lower transverse relaxation and distribution compared to *Fth1-*BMSCs, and the reason needs further exploration. A comparison of the extent of iron aggregation in the infarction lesions showed that *Fth1*-BMSCs accumulated more iron than SPIO-BMSCs under stroke condition. This was reflected in the SWI sequence *in vivo*, which showed a lower signal contrast compared to the control group. Prussian blue staining and pathological observation showed that a high density of blue-stained iron particles accumulated around the infarction area and grew into the infarction center in the *Fth1-*BMSCs group, which suggested that *Fth1*-labeled BMSCs could take up iron particles from the surrounding tissue.

SWI clearly showed hypointense lesions in the SPIO-BMSC group (only traceable for 30 d) and the *Fth1-*BMSC group. T2WI showed hypointense lesions for only 20 d in the SPIO-BMSC group. T2WI was not sensitive enough for the *Fth1-*BMSC group. This indicated that the intracellular iron concentration of *Fth1*-labeled BMSCs was equivalent to the intracellular iron concentration of the 50 *μ*g/ml SPIO-labeled BMSCs on the 20^th^ day. Compared to the T2WI sequences, SWI sequences show a longer and a wider range of hypointense signals. Therefore, SWI is more sensitive and can be chosen as the optimal MRI-tracking sequence.

*Fth1-*BMSCs maintain the same signal intensity between the 10^th^ and the 60^th^ day and show a similar distribution range between the 40^th^ and the 60^th^ day. Although current medical imaging technologies restricted us from detecting very low concentrations of iron, we can exclude that the *Fth1-*BMSCs multiplied and migrated around the lesion; that is, mitosis of BMSCs may have stopped, and committed differentiation might have occurred. Hence, it is possible to observe the proliferation and differentiation of stem cells by the *Fth1*-labeling method [15, 27, 29]. The *Fth1*-labeling method was stable and could be used for long-term tracking.

## 4. Conclusions

In summary, *Fth1* coupled to a shuttle plasmid (pCDH-CMV-MCS-EF1-copGFP) displays satisfactory properties for utilization as an MRI reporter gene for *in vivo* detection of BMSCs transplanted in response to cerebral ischemia reperfusion injury and used for therapeutic interventions. The *Fth1*-labeling method, based on a standard clinical MRI, can potentially prove to be a powerful tool for the combined, noninvasive evaluation of molecular activity of transplanted cells and is stable and suitable for long-term tracking of cells. Compared to T2WI, SWI is more sensitive and can be chosen as the optimal MRI-tracking sequence.

In this study, we provided a proof of the concept that a molecular probe constituting the *FTH* gene can be utilized for the detection of the chemotaxis and migration of BMSCs by MRI. In addition, we could also document a marked beneficial effect of *Fth1-*BMSC administration; however, specifically designed studies will be necessary to further characterize the therapeutic actions of BMSCs on cerebral ischemia reperfusion injury models. Further work will be necessary to test the reliability of this methodology for tracking changes over the entire process of postinfarct remodeling and under other physiological and pathological conditions. In addition, more research is required to study the long-term safety and differentiation properties of *Fth1-*BMSCs in rat models.

## Figures and Tables

**Table 1:** Fluorescence quantitative real-time PCR results of control and experimental groups.

Sample description	*β*-actin	*Fth1*	ΔCT	ΔΔCT	2^−ΔΔCT^
Control	18.52	28.01	9.49	0	1
*Fth1*	18.12	23.49	5.37	−4.11	17.34

Note: the quantitative assay of each sample was conducted thrice; using internal parameters, the relative quantities of mRNA were calculated using the 2^−ΔΔCT^ algorithm. 2^−ΔΔCT^ represents the consequence. By the standard of cells responding to EGFP-*Fth1* viral infection, 2^−ΔΔCT^ was calculated.

**Table 2:** Absorbance values of experimental and control groups 48 h after transfection.

Group	OD value $\left(\bar{x}\pm \text{s}\right)$	*p* value
Control	1.2289 ± 0.0367	<0.05
*Fth1*-BMSC	1.2821 ± 0.0253	<0.05

Note: OD: optical density; *p* < 0.05, if there are statistical differences.

## Data Availability

All data generated or analyzed during this study are included in this published article.

## Supplementary Materials

Supplementary Materials Supplemental Figure 1: vector plasmid profile and multiple cloning site information of (A) pCDH-CMV-MCS-EF1-copGFP-*Fth1*, (B) psPAX2, and (C) pMD2.G. Supplemental Figure 2: results of BMSC surface antigens CD90, CD44, and CD34 detected by flow cytometry. Supplemental Figure 3: results of staining with Alizarin Red and Oil Red O 21 d after osteoblastic (A) and adipogenic (B) induction of BMSCs. Supplemental Figure 4: results of fluorescence microscopy after EGFP gene expression in *Fth1*-BMSCs for 48 h and further culturing for 3-4 d ((A) in normal light and (B) in fluorescent light). Supplemental Figure 5: results of FTH1 protein expression in the control group and *Fth1*-BMSC group.

## References

[1] Kim H.-J., Jung J., Park J.-H., Kim J.-H., Ko K.-N., Kim C.-W. (2013). Extremely low-frequency electromagnetic fields induce neural differentiation in bone marrow derived mesenchymal stem cells. *Experimental Biology and Medicine*.

[2] Jiang W., Liang G., Li X. (2014). Intracarotid transplantation of autologous adipose-derived mesenchymal stem cells significantly improves neurological deficits in rats after MCAo. *Journal of Materials Science: Materials in Medicine*.

[3] Pourheydar B., Soleimani Asl S., Azimzadeh M., Moghadam A. R., Marzban A., Mehdizadeh M. (2016). Neuroprotective effects of bone marrow mesenchymal stem cells on bilateral common carotid arteries occlusion model of cerebral ischemia in rat. *Behavioural Neurology*.

[4] Polymeri A., Giannobile W., Kaigler D. (2016). Bone marrow stromal stem cells in tissue engineering and regenerative medicine. *Hormone and Metabolic Research*.

[5] Tarulli E., Chaudhuri J. D., Gretka V., Hoyles A., Morshead C. M., Stanisz G. J. (2013). Effectiveness of micron-sized superparamagnetic iron oxide particles as markers for detection of migration of bone marrow-derived mesenchymal stromal cells in a stroke model. *Journal of Magnetic Resonance Imaging*.

[6] Shim J., Kwak B. K., Jung J., Park S. (2015). Evaluation of engraftment of superparamagnetic iron oxide-labeled mesenchymal stem cells using three-dimensional reconstruction of magnetic resonance imaging in photothrombotic cerebral infarction models of rats. *Korean Journal of Radiology*.

[7] Lee H. N., Ko K. N., Kim H. J., Rosebud Aikins A., Kim C. W. (2015). Ferritin is associated with neural differentiation of bone marrow-derived mesenchymal stem cells under extremely low-frequency electromagnetic field. *Cellular and Molecular Biology (Noisy-le-Grand, France)*.

[8] Zhao S., Wang Y., Gao C. (2014). Superparamagnetic iron oxide magnetic nanomaterial-labeled bone marrow mesenchymal stem cells for rat liver repair after hepatectomy. *Journal of Surgical Research*.

[9] Feng Y., Liu Q., Zhu J., Xie F., Li L. (2012). Efficiency of ferritin as an MRI reporter gene in NPC cells is enhanced by iron supplementation. *Journal of Biomedicine and Biotechnology*.

[10] Kim H. S., Woo J., Choi Y. (2015). Noninvasive MRI and multilineage differentiation capability of ferritin-transduced human mesenchymal stem cells. *NMR in Biomedicine*.

[11] Yang Y., Gong M.-F., Yang H. (2016). MR molecular imaging of tumours using ferritin heavy chain reporter gene expression mediated by the hTERT promoter. *European Radiology*.

[12] Cheng S., Mi R., Xu Y. (2017). Ferritin heavy chain as a molecular imaging reporter gene in glioma xenografts. *Journal of Cancer Research and Clinical Oncology*.

[13] He X., Cai J., Liu B., Zhong Y., Qin Y. (2015). Cellular magnetic resonance imaging contrast generated by the ferritin heavy chain genetic reporter under the control of a tet-on switch. *Stem Cell Research and Therapy*.

[14] Stuckey D. J., Carr C. A., Martin-Rendon E. (2006). Iron particles for noninvasive monitoring of bone marrow stromal cell engraftment into, and isolation of viable engrafted donor cells from, the heart. *Stem Cells*.

[15] Song C., Wang J., Mo C. (2015). Use of ferritin expression, regulated by neural cell-specific promoters in human adipose tissue-derived mesenchymal stem cells, to monitor differentiation with magnetic resonance imaging in vitro. *PLoS One*.

[16] Naumova A. V., Yarnykh V. L., Balu N., Reinecke H., Murry C. E., Yuan C. (2012). Quantification of MRI signal of transgenic grafts overexpressing ferritin in murine myocardial infarcts. *NMR in Biomedicine*.

[17] Iordanova B., Ahrens E. T. (2012). *In vivo* magnetic resonance imaging of ferritin-based reporter visualizes native neuroblast migration. *NeuroImage*.

[18] Moisan A., Pannetier N., Grillon E. (2012). Intracerebral injection of human mesenchymal stem cells impacts cerebral microvasculature after experimental stroke: MRI study. *NMR in Biomedicine*.

[19] Lobello N., Biamonte F., Pisanu M. E. (2016). Ferritin heavy chain is a negative regulator of ovarian cancer stem cell expansion and epithelial to mesenchymal transition. *Oncotarget.*.

[20] Hu J., Liu Y., He M., Yu B., Wang G. (2013). Recombinant human bone morphogenetic protein-2 down-regulates LASP1 and up-regulates ferritin during osteogenic differentiation of beagle dog bone marrow mesenchymal stem cells. *Nan Fang Yi Ke Da Xue Xue Bao*.

[21] Dai H. Y., He R., Zhang Y., Wu R. H., Xiao Y. Y. (2017). Adenoviral vector mediated ferritin over-expression in mesenchymal stem cells detected by 7T MRI in vitro. *PLoS One*.

[22] Cao M., Mao J., Duan X. (2018). *In vivo* tracking of the tropism of mesenchymal stem cells to malignant gliomas using reporter gene-based MR imaging. *International Journal of Cancer*.

[23] Correia Carreira S., Armstrong J. P. K., Seddon A. M., Perriman A. W., Hartley-Davies R., Schwarzacher W. (2016). Ultra-fast stem cell labelling using cationised magnetoferritin. *Nanoscale*.

[24] Pereira S. M., Moss D., Williams S. R., Murray P., Taylor A. (2015). Overexpression of the MRI reporter genes ferritin and transferrin receptor affect iron homeostasis and produce limited contrast in mesenchymal stem cells. *International Journal of Molecular Sciences*.

[25] Valero E., Fiorini S., Tambalo S. (2014). *In vivo* long-term magnetic resonance imaging activity of ferritin-based magnetic nanoparticles versus a standard contrast agent. *Journal of Medicinal Chemistry*.

[26] Ruan G.-P., Han Y.-B., Wang T.-H. (2013). Comparative study among three different methods of bone marrow mesenchymal stem cell transplantation following cerebral infarction in rats. *Neurological Research*.

[27] Balogh E., Tolnai E., Nagy B. (2016). Iron overload inhibits osteogenic commitment and differentiation of mesenchymal stem cells via the induction of ferritin. *Biochimica et Biophysica Acta (BBA)-Molecular Basis of Disease*.

[28] Tang H. L., Sun H. P., Wu X., Sha H. Y., Feng X. Y., Zhu J. H. (2011). Detection of neural stem cells function in rats with traumatic brain injury by manganese-enhanced magnetic resonance imaging. *Chinese Medical Journal (Engl).*.

[29] Guo R., Li Q., Yang F. (2018). *In vivo* MR imaging of dual MRI reporter genes and deltex-1 gene-modified human mesenchymal stem cells in the treatment of closed penile fracture. *Molecular Imaging and Biology*.

