# Supplementary material for: MRI Tracking of SPIO- and Fth1-Labeled Bone Marrow Mesenchymal Stromal Cell Transplantation for Treatment of Stroke
Source: Contrast Media Mol Imaging. 2019 Aug 29;2019:5184105. doi: 10.1155/2019/5184105 (PMC6735219; doi:10.1155/2019/5184105)
Supplement: Supplementary Materials — Supplemental Figure 1: vector plasmid profile and multiple cloning site information of (A) pCDH-CMV-MCS-EF1-copGFP-Fth1, (B) psPAX2, and (C) pMD2.G. Supplemental Figure 2: results of BMSC surface antigens CD90, CD44, and CD34 detected by flow cytometry. Supplemental Figure 3: results of staining with Alizarin Red and Oil Red O 21 d after osteoblastic (A) and adipogenic (B) induction of BMSCs. Supplemental Figure 4: results of fluorescence microscopy after EGFP gene expression in Fth1-BMSCs for 48 h and further culturing for 3-4 d ((A) in normal light and (B) in fluorescent light). Supplemental Figure 5: results of FTH1 protein expression in the control group and Fth1-BMSC group. [file 5184105.f1.zip › 5184105.f1/Supplemental Figure 4_CMMI_2807705.doc]

Supplemental Figure 4

A

B
